# Three-Dimensional Magnetic Induction Tomography: Practical Implementation for Imaging throughout the Depth of a Low Conductive and Voluminous Body

**DOI:** 10.3390/s21227725

**Published:** 2021-11-20

**Authors:** Martin Klein, Daniel Erni, Dirk Rueter

**Affiliations:** 1 Institute of Measurement Engineering and Sensor Technology, University of Applied Sciences Ruhr West, D-45407 Mülheim an der Ruhr, Germany; dirk.rueter@hs-ruhrwest.de; 2 General and Theoretical Electrical Engineering (ATE), Faculty of Engineering, University of Duisburg-Essen, and CENIDE—Center for Nanointegration Duisburg-Essen, D-47048 Duisburg, Germany; daniel.erni@uni-due.de

**Keywords:** magnetic induction tomography, electromagnetic tomography, three-dimensional imaging, biomedical imaging, 3D reconstruction

## Abstract

Magnetic induction tomography (MIT) is a contactless, low-energy method used to visualize the conductivity distribution inside a body under examination. A particularly demanding task is the three-dimensional (3D) imaging of voluminous bodies in the biomedical impedance regime. While successful MIT simulations have been reported for this regime, practical demonstration over the entire depth of weakly conductive bodies is technically difficult and has not yet been reported, particularly in terms of more realistic requirements. Poor sensitivity in the central regions critically affects the measurements. However, a recently simulated MIT scanner with a sinusoidal excitation field topology promises improved sensitivity (>20 dB) from the interior. On this basis, a large and fast 3D MIT scanner was practically realized in this study. Close agreement between theoretical forward calculations and experimental measurements underline the technical performance of the sensor system, and the previously only simulated progress is hereby confirmed. This allows 3D reconstructions from practical measurements to be presented over the entire depth of a voluminous body phantom with tissue-like conductivity and dimensions similar to a human torso. This feasibility demonstration takes MIT a step further toward the quick 3D mapping of a low conductive and voluminous object, for example, for rapid, harmless and contactless thorax or lung diagnostics.

## 1. Introduction

The contactless method of magnetic induction tomography (MIT) has attracted interest for over two decades due to its harmlessness and the simple generation and detection of low-energy induction fields. These fields easily permeate free space and all non-ferromagnetic materials at sufficiently low conductivity. The method of application is relatively straightforward. Primary induction coils induce electric fields in the required measuring range, whereby eddy currents are established within the conductive body under examination. The conductivity distribution of the body shapes the eddy currents, and their secondary induction field is detected by receiver coils. The stronger primary field would also be detected by the receivers, but this can be avoided by using various techniques, such as backoff coils [1] or gradiometry [2,3]. Finally, a computer translates the signals obtained into a visualization of the conductivity distribution under investigation.

With typical annular MIT setups, the sensitivity in the central areas of weakly conductive measurement objects (i.e., voluminous bodies) has so far been very low [3,4,5,6,7]. However, with a new type of MIT setup [8], simulations have shown that a sensitivity increase of more than 20 dB can be achieved. This has enabled the 3D imaging of conductivity changes in the center of a conductive volume. Here, this novel MIT method is comprehensively demonstrated in an experimental setting for the first time. Thereby, low conductivities (0 to 1 S/m) in the biomedical range are accessible with this novel approach.

In general, biomedical MIT applications have appeared attractive for a long time. Other contactless imaging methods include magnetic resonance imaging (MRI), which typically uses a strong and stable magnetic field, with different gradients for spatial encoding and radio frequency (RF) for excitation and detection, and X-ray-based computed tomography (CT). Compared with these, MIT appears to offer a relatively fast, convenient, and generally harmless whole-body tomography method. Moreover, the potential system cost of MIT is low compared with MRI and CT. The material costs for this experimental setup are up to 3000 €. Furthermore, the method is still contactless in contrast to sonography or electrical impedance tomography (EIT). However, MIT also has significant inherent drawbacks and weaknesses that make the method difficult and ultimately result in only modest resolution. Since the induction fields quickly become weaker and blurred with increasing distance, there is an information loss, particularly for those areas distant from the inductors, resulting in an ill-posed inverse problem. Therefore, MIT requires a precise measurement technique and high computational effort. Nevertheless, the rather low resolution could be sufficient to resolve centimeter-sized features inside a body. This could be sufficient for various applications, for example, to localize major lung anomalies or mass bleeding inside the torso.

Similar to scintigraphy, which is also low-resolution, MIT offers an alternative contrast method, namely, the distribution of electrical conductivity. This could be used, for example, in non-invasive mapping of the conductivity of cardiac tissue [9]. The MIT approach presented in our article is not intended to compete with or replace existing, well developed tomography methods. Thus, it is not the replacement of, but the potential addition to, existing methods that makes MIT particularly relevant. More biomedical applications might arise in the field of rapid, non-hazardous, non-contact lung diagnostics [10], or in the rapid detection of hemorrhage in the human skull [11]. Nevertheless, possible applications are not limited to biomedicine. An MIT setup could also serve as a quick, non-contact body scanner at security gates, for example, at airports, where currently implemented systems for harmless mass application can either detect metallic objects (metal detector gates) or are essentially restricted to the body surface (terahertz scanner). In contrast, MIT could resolve non-metallic and centimeter-sized objects of concern inside the body, without critical energies such as those of X-rays or the strong electromagnetic fields of MRIs. Many industrial applications are also being discussed. Alongside previous work on metallic targets, where the signal response is stronger than for the low conductivity regime in biomedicine, multiphase mixtures or flows can be analyzed with tomography systems, allowing the imaging of the spatial distribution of substances [12].

However, this article does not address a specific application, it is only about advancing basic research in the field of MIT. For this reason, the practical implementation of 3D MIT has been considered here for the first time, to detect and localize perturbations throughout the depth of a weakly conductive (“biomedical”) test body. With the exciter and receiver geometries previously used [3,4,5,6,7,13,14,15,16,17,18,19,20,21,22,23], the sensitivity in the central areas of a weakly conductive body is critically low [3,4,5,6,7]. In addition to the attenuation and blurring of the induction field over distance, there is another effect that further reduces the sensitivity at the center of the body: the induced eddy currents run in closed loops within the body, and the current density thus tends to be strongest near the surface, but decreases toward the center region (and along the axis of the coils), where it vanishes and no longer provides any information [8]. Therefore, only simulations of biomedical 3D MIT have been successful in resolving weakly conductive inhomogeneities at the center of a test body to this point [8,24,25]. Yet, MIT simulations can be too optimistic, as they can be based on idealized prerequisites that are difficult to implement in practice.

Therefore, for initial biomedical MIT realizations, various evasion methods were used so far to ensure that the signals received were usable. The perturbations were either placed close to the surface of the test body; were centered but breaking through the lower and upper surface of a shallow bath (a quasi-2D setup) while being partly isolated from the conductive background by plastic containers; were not immersed in a conductive background; or were not weakly conductive, but instead metallic. Usually, the perturbations had a relative volume (RV) from 3% to 8% of the total volume of the body [6,15,18], which corresponds to a rather poor resolution. In addition, the measurement sequence typically required more than a few seconds, thus causing the method to be uncomfortable or even unfeasible for living beings, especially because of the high susceptibility of MIT to motion artifacts [26].

A more realistic biomedical MIT approach requires the simultaneous fulfillment of several restrictive requirements for the phantoms and the measurement: (i) the conductivities for the background (body) and sought feature (perturbation) should be in the range of biological tissues (i.e., from 0 S/m to 2 S/m [27]); (ii) the body should have representative dimensions, for example, of the human torso or head; (iii) the sought perturbation should be relatively small in relation to the whole body, such as a deviation in lung density or mass hemorrhage in relation to the human torso; (iv) the perturbation should be continuously interconnected to the surrounding background, and no plastic insulation should be placed around a conductive perturbation; (v) the perturbations should be detectable throughout the depth of the body; they should not be located only near the surface of the volume, where the signals are much stronger; (vi) the entire problem must be treated technically and mathematically in 3D; in principle, a 2D approach cannot satisfy voluminous, biomedical MIT because the eddy current extends into a volume; and (vii) the measurement data must be acquired within seconds, otherwise, motion artifacts from living beings will prevent sufficient quality.

These more realistic requirements were recently addressed [8], whereby a wave-shaped (undulating) excitation field was used. Intentionally increased eddy current densities in the center of a test volume provided a sensitivity gain of more than 20 dB in the central areas compared with annular MIT setups with circular coils, making the depth of the volume more easily accessible. Using simulations, the principles and inherent advantages of the undulating excitation field were extensively discussed in that previous study. At that time, the reported technique was not ready for multichannel acquisition in 3D, and a more extended undulator to create a virtually sinusoidal field in the measuring range of the receivers had not been implemented. Brief experimental verification was achieved using a reduced undulator, consisting of just five strips, and only one receiver. The undulator did not provide a sufficiently pure sinusoidal field and the whole setup was insufficient for practical 3D MIT results. Although the practical measurement signals only showed a rough approximation to the simulated measurements, this similarity was insufficient for the MIT calculation. Therefore, in the previously shown 3D reconstructions based on simulated data, experimental evidence of truly practical feasibility was ultimately not provided. As mentioned above, simulated MIT successes often fail to materialize in practice because real circumstances are not considered or are only considered in an idealized way.

Here, a multichannel 3D MIT scanner with an extended undulator is experimentally demonstrated. Particular care is taken to ensure that the previously described criteria for realistic biomedical MIT verification are met. The practical design enables the rapid detection of small (less than 2% RV), and weakly conductive (0–1 S/m) perturbations in a weakly conductive background (0.5 S/m) with dimensions similar to a human torso. However, the necessary agreement between the forward model and the measured signals required for successful reconstruction poses a great challenge to experimental MIT. Systematic signal deviations originate from many different sources, for example, imperfections in coil implementation, any geometrical mismatch between the forward model and the real setup, additional coupling paths (capacitive coupling), or effects caused by inappropriate voxel size [20]. In addition, both the input signals for the exciter and the reference at the receiver must be stabilized in amplitude and phase. Furthermore, environmental vibrations cannot be considered in the forward model, and such dislocation noise (and other noise sources) has to be reduced as much as possible.

## 2. Materials and Methods

### 2.1. Applied Techniques for 3D MIT Imaging

The MIT setup implemented practically here approaches the setup only simulated in [8]. In order to achieve 3D reconstructions based on real measurements, all feasible aids were exploited, which are listed below:

- Virtually perfect gradiometry was established. The receivers were aligned gradiometrically with the transmitter field; thus, almost all received signals originated in the measurement object itself [2,3]. This means that the full dynamic range of the receiver can be used for the secondary field, that is, the imprint of the test body of interest.
- Measurements were made using transmission geometry, which means that the test body was mainly located between the excitation coil and the receiver coils. Thus, the fields always passed through the entire body. This technique increased the amount of information about the interior of the body.
- Due to the rapidly blurring and decaying induction fields over distance, the geometric gap between the excitation and receiver coils was kept as small as possible. Consequently, the smallest dimension of the test body ultimately determined the required gap [8]. Preferred directions can, and should, therefore be used to facilitate MIT challenges. For example, the human torso is not normally spherical in shape.
- A Weak coupling approach was considered. The operating frequency was chosen to be so low (here: 1.5 MHz) that the induction fields would not experience significant attenuation or distortion in the weakly conducting test body. This allowed the assumption that the primary field was not affected or altered inside the body, which considerably simplified the calculation [28].
- A single, planar exciter (undulator) provided the wave-shaped primary field, which enhanced the central area sensitivity (>20 dB) together with the lateral scan procedure.
- The lateral and linear movement of the test object between the excitation coil (undulator) and the opposing receivers provided a considerable amount of independent data within 10 s. The mechanical scan was subjected to low mechanical vibration and motion artifacts.
- In forward modeling, the eddy currents in the object have to be recalculated for each position of the lateral scan method (here, 200 x-positions). However, the frequently performed, computationally expensive forward problem was significantly reduced using a sinusoidal field topology. Only two eddy current solutions had to be calculated, and the total MIT computation was accelerated by one order of magnitude [8]. This advantage required an extended undulator for only one significant spatial frequency in the x-direction. The previously applied and more compact excitation with only five strips could not provide a sufficiently clean sinusoidal field topology.
- Butterfly receivers were used instead of circular receiver coils. In comparison to a circular receiver coil, this geometry further increased the sensitivity of the dipole-shaped current fields typically originating from local perturbations in a conductive background (by about 6 dB).
- As a practical simplification, a restriction was initially made to use only voluminous cuboids with torso-like dimensions. The general functionality of the system was, however, not restricted to voluminous cuboids. Although possible, the acquisition and modeling of arbitrarily shaped bodies is more complex and initially more prone to errors.

### 2.2. Electro-Mechanical MIT Scanner Setup

**Figure 1:**
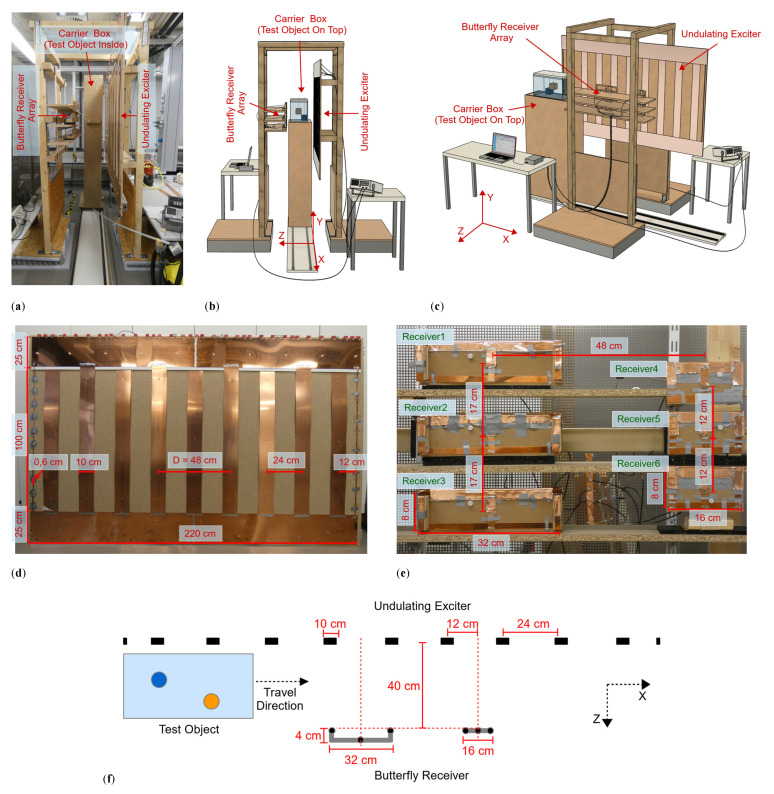
Planar MIT scanner setup. (**a**) Practical realization; (**b**,**c**) Schematic illustration, the upper half of the carrier box is omitted for better visualization of the test object; (**d**) Undulating exciter with dimensions shown, consisting of 11 copper strips with antiparallel current directions. The distance *D* describes the periodicity of similar structures and currents; (**e**) Butterfly receiver array with dimensions. The left column contains the wide receivers, and the right column contains the narrow receivers; and (**f**) Exciter and receiver alignment in cross-section from the central plane.

The practical implementation of the planar MIT setup (Figure 1a–c) was created with dimensions suitable for scanning a human being; it consisted of three functional modules.

An undulating excitation coil (undulator) projected a well-balanced and steady-state induction field at 1.5 MHz. An opposing receiver array consisted of six butterfly coils, which were gradiometrically aligned in the excitation field, such that the six idle signals were virtually zero with respect to the imprint of the test body. The test object was housed in a carrier box and moved laterally in the x-direction on a 300 cm-long rail system through a 40 cm gap (extending in the z-direction) between the exciter and receiver. A practical scan was completed in 10 s, and each receiver took a measurement value every 1 cm along the active, 200 cm-long, central scanning path. The recorded dataset thus equaled 200 × 6 = 1200 measurements.

The undulator (Figure 1d) behaved like a single LC parallel circuit with a resonance frequency of 1.5 MHz, whereby a relatively modest driving power at the resonance frequency resulted in considerably enhanced currents in the copper strips. The 1.5 MHz induction field was only slightly absorbed or deformed by the weakly conductive test object; thus, it could be advantageously treated as a weakly coupled problem [29]. Furthermore, the power introduced into the test object could always be guaranteed to be less than the driving power (less than 2 W), making this a harmless method with low power density in the measurement volume. The undulator consisted of 11 elongated, equidistant copper strips vertically aligned in the y-direction (Figure 1d). The strip currents ran in alternating directions (periodicity *D* = 48 cm) and at equal amplitudes. Only the two outermost strips were smaller and carried half the current. The primary field in the design had a periodically repeating vector potential (isomorphic to the induced electrical field), which in the measurement volume was directed only in the y-direction. The approximately sinusoidal shape of the field in the x-direction was preserved in the area under investigation rather than widening with depth z [8]. As a consequence, the field intensity exponentially decayed with depth in the z-direction. For a primary of periodicity *D*, the characteristic eddy current loops inside the voluminous test body cannot typically be wider than *D*/2 in the x-direction. The eddies must then pass through the central volume of wide objects. Therefore, the eddy current density here was higher on average throughout the object compared with typical MIT excitation by circular coils [3,4,5,6,7,13,14,15,16,17,18,19,20,21,22,23]. Improved central area sensitivity (>20 dB) was the result of this spatial prefiltering [8]. Central perturbations became much more detectable above the noise floor, and the relative imprint of minor dislocations or mechanical vibrations in the receiver signals was accordingly reduced.

In addition, because of their butterfly geometry, the six receivers (Figure 1e,f) achieved an increased sensitivity of approximately 6 dB [8] in dipole-shaped current fields compared to circular receiver geometries. The dipole-shaped current fields were typically emitted by local perturbations in the conductive background determined by the biomedical application. The receivers were arranged in two vertical columns with three narrow and three wider receivers. The depth range (i.e., the z-range) of the two geometries differed, thus allowing for better depth resolution in the MIT. Due to their geometry, the vector potential of the narrow receivers was larger in the vicinity of these receivers than at deeper points of the measuring range, so that their detection range also tended to be nearer the receivers. The wide receivers achieved a greater depth range and were modified so that their vector potential (i.e., relevant y-component) crossed zero just beyond the object boundary near the exciter. In addition, the outermost wires of the wide butterfly receivers (considering the y-direction) were elevated in the z-direction toward the exciter (Figure 1f). This modification partly compensated for the strong currents in the y-direction in the object near the exciter, which tended to deliver disproportionately strong signals from this region. The resolution in the z-direction was obtained by the signal shape but was also improved by the different receiver sizes previously described. Resolution in the x-direction was implicitly provided by the scan in the x-direction, and in the y-direction by the arrangement of the receivers in vertical columns. Shielding the receivers reduced capacitive coupling between the exciter, the test object and the receiver, which otherwise might have become a major issue [30] in the biomedical regime where phantoms or bodies are rich in water (low conductivity and high dielectric constant). In addition, there is a slight coupling among the receivers. Despite this coupling, the difference signals considered here are sufficient for a 3D reconstruction. For future work, especially for reconstructions with total signals, this coupling must be considered and revised separately.

The two receiver columns were located opposite the transmitter with the test body placed between them (i.e., transmission geometry), and they were both centered between two undulator strips. Due to the elementary symmetry, the primary field was almost completely canceled in the received signals (i.e., gradiometric alignment, [2,3,8,31]). The six 1.5 MHz signals received were amplified and directly mixed down to DC according to a reference signal from the excitation site. The DC measurement value changed with each step along the 200 cm-long scanning path. This value was digitized (Arduino Due) and transmitted to the computer accompanied by the scanning position discretized in 1 cm steps. The phase position of the signal depended on the object under investigation. For example, signals from resistive materials had a phase angle of about 90° to those from inductive materials (highly conductive metals). By phase tuning the reference signal at the mixer, these different regimes became directly accessible. Overall, the system was critically affected by phase drift and noise. For example, temperature-dependent capacitors of the exciter oscillating circuit caused a slow phase drift, which affected the signal stability. The transmitter LC circuit was thus damped with resistors, resulting in a flattened phase response.

The complete electro-mechanical MIT scanner setup was installed in a normal laboratory environment that had many sources of quasi-seismic or mechanical interference (e.g., people walking somewhere in the building, opening and closing doors, etc.). The effect with the greatest influence was that of mechanical vibrations, which disturbed the relative positioning between the exciters and receivers, thereby inducing the largest artifacts and noise. To mitigate this, the entire transmitter and receiver assembly were damped with foam mats and springs as well as being stiffened against each other. In addition, the rails and, thus, the moving carrier box were completely detached from the rest of the setup.

### 2.3. Conductive Body Phantom

**Figure 2:**
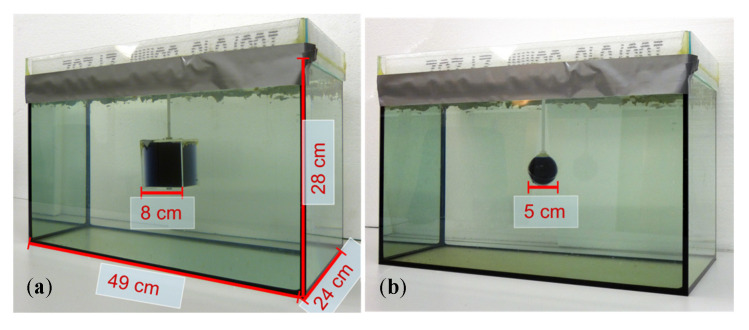
The 33-L saline body phantom with a non-conducting perturbation centered in the horizontal middle plane. Body dimensions: 49 cm in the x-direction, 28 cm in the y-direction, and 24 cm in the z-direction. (**a**) Body with cuboidal perturbation (512 mL); (**b**) Body with spherical perturbation (65 mL).

The test body (Figure 2), the dimensions of which were similar to a human torso (49 cm × 28 cm × 24 cm), was a 33-L saline bath (0.5 S/m). The largest dimension (49 cm) extended in the x-direction and thereby approached the undulator periodicity *D*. Greater length in the x-direction would not have affected the underlying principles. The smallest body dimension (24 cm) was extended in the most sensitive direction, that is, the z-direction (the gap between excitation and receiving). The smaller this gap, the higher the potential resolution of the MIT. This is similar to the obsolete tape recorder, where the undesired air gap between the audio tape and the magnetic head had to be as small as possible to reproduce high audio frequencies, corresponding to smaller and more densely arranged magnetic structures in the tape.

A polystyrene covering plate inhibited the random motion of the water (i.e., motion artifacts) during a scan. The introduced perturbations were steady with respect to the traveling saline bath, and their effective conductivities (either 0 or 1 S/m) were in the typical range of human organs or tissues: 0 S/m is similar to fat tissue, a pneumothorax, or highly aerated lungs, and 1 S/m is similar to free blood or serum. For the 3D reconstructions (Section 3.2), cuboidal perturbations were used (Figure 2a). The cuboidal perturbations that were placed in various positions within the body provided a RV of 1.55% (8 cm × 8 cm × 8 cm = 0.512 L) with respect to the 33-L saline cuboid. The smallest clearly detectable perturbation in the interior of the volume was a sphere with a diameter of 5 cm (65 mL, 0.2% RV; Figure 2b).

**Figure 3:**
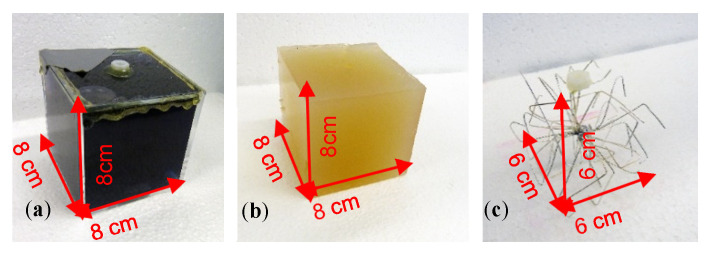
Cuboidal perturbations corresponding to a 0.512-L (RV = 1.55%) feature. (**a**) Plastic cube filled with deionized water (0 S/m); (**b**) Saline-agar cube (1 S/m); (**c**) Wire star in a conductive background resulted in virtually the same signal as that obtained by the saline-agar cube (1 S/m).

Perturbations at 0 S/m consisted of a plastic cube or sphere filled with deionized water (Figure 3a). To ensure a realistic representation of the situation in living beings, all sides of the more conductive perturbation were connected to the conductive environment; thus, it was not isolated by a plastic shell. More conductive perturbations were realized using saline-agar cubes (1 S/m; Figure 3b). Although this material has been established for application in biomedical impedance phantoms, being easy to shape, it is, however, not durable. As this material is organic, it dissolves and/or decomposes over time, thus hindering experiments conducted over longer periods. A virtually equivalent replacement for the saline-agar perturbation was found in a much more durable wire star (Figure 3c) shaped from multiple, open-ended copper wires (silver coated).

Due to the open-ended wires, the eddy current loops must be closed by the weakly conductive background of the environment. Furthermore, it was ensured that this wire star reproduced the differential MIT signals of the 1 S/m saline-agar cube within the 0.5 S/m background. For this purpose, measurements were made with both the saline-agar cube and the wire star at different locations in the 33-L body. The comparison showed that the signal curves and amplitudes were quite similar, and the phase was virtually the same (Appendix A). Only small-sized eddies occurred inside the open-ended but highly conductive wires, at a relative phase shift of approximately 90°, which was mostly undetected in the phase-locked receiver circuit. The open-ended wire star immersed in the weakly conducting background did not therefore appear as a highly conductive solid metal object. It was therefore used as a durable replacement for the established but unstable saline-agar cube (1 S/m).

The test body and the introduced perturbations described here fulfilled the requirements for a realistic biomedical MIT, as described in the introduction.

### 2.4. Reconstruction in the Computer

In 3D MIT, the computational effort required is high [32], a major portion of which is caused by the nonlinear response to conductivity variations. The conductivity was typically approximated by numerous recalculations using iterative approaches.

In the reconstruction, it was necessary to set up a forward model that simulated and calculated the physical signal chain from the excitation coils, over the body to the receivers, resulting in simulated measurements. The forward model was calculated using the generalized version of Geselowitz’s relationship [33] based on the reciprocity theorem, that is, the integration of the inner product of the virtual receiver field (represented by the vector potential) and the calculated eddy current density field in the object representing the receiver signal [34]. The virtual receiver field remained constant and had to be calculated only once. However, the eddy current density field (J) throughout the object had to be recalculated for each position (x) along the scanning path, and for this reason is referred to as $J_{x}$. This recurring calculation accounted for a major fraction of the total computational cost. The undulator used here allowed for considerably accelerated calculation of the eddy current density field by intentionally generating the periodic and even sinusoidally-shaped excitation with periodicity D (Figure 1d), as shown in Equation (1):

(1) $$J_{x}=J_{\Phi }\cos\left(\frac{2\pi x}{D}\right)+J_{\Psi }\sin\left(\frac{2\pi x}{D}\right),$$

where $J_{\Phi }$ corresponds to the eddy current density field throughout the object when the object is centered exactly in front of the middle exciter copper strip and $J_{\Psi }$ corresponds to the eddy current density field when the object moves by D/4 and is therefore exactly between two strips. Hence, only two eddy current solutions ($J_{\Phi }$ and $J_{\Psi }$) had to be calculated, and their weighted superposition revealed $J_{x}$ at any scan position [8]. The reconstructions presented here are currently based on differential measurements because the computer-aided and idealized forward model, although it performed well, was still not exact enough to describe the total signals measured in practice, as shown in the experimental results below. Instead of MIT using absolute measurements, differential measurements of local perturbations were utilized because they were already a satisfactory match to the differential forward calculation. The reconstruction algorithm (implemented in MATLAB R2021a) corresponded to that of our previously published procedure [8], where an iterative algorithm based on the inverted and regularized Jacobian was used to reconstruct the practical 3D conductivity distribution inside the voluminous body. Direct inversion in a single step was hampered by the high complexity of the generally nonlinear MIT problem.

The authors are aware that more mature theories and algorithms will solve the problems better and faster. Such theories and algorithms will be implemented in our institute in the near future by a team of professional mathematicians; a specific project to address these issues has recently been approved. The scope of this paper, though, is the practical progress of 3D MIT throughout a volume within the realistic requirements of a biomedical application.

## 3. Experimental Results

### 3.1. Measurement Signal Validation

The practical setup presented here is intended to verify whether local differences in conductivity can be detected in the central regions of a voluminous body and whether the sensitivity from the central regions is similar to that near the surface. In addition, it examines how well the practical signals match the predictions from the forward calculation. For successful reconstruction, high correspondence between these signals is necessary.

**Figure 4:**
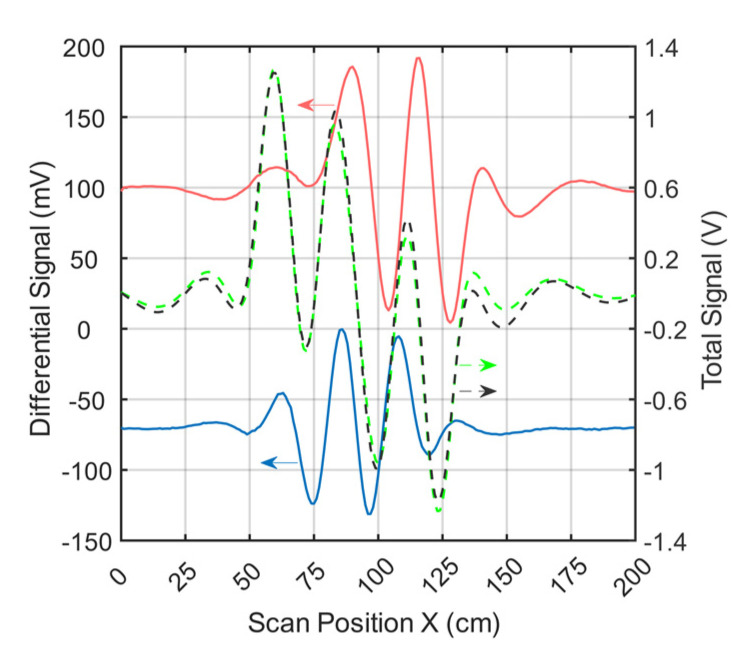
Differential and total signals (receiver 2). The arrows point to the relevant y-axis. The dotted black line represents the measured total signal of the body; the dotted green line is the simulated total signal; the red line is the measured differential signal of a perturbation when face-centered at the side wall; and the blue line is the measured differential signal of a perturbation in the center of the body.

Figure 4 shows the signal trace of receiver 2 (Figure 1e) for a perturbation (0.512 L at 0 S/m) immersed within the conductive background (33 L at 0.5 S/m; cf., Figure 2a). The perturbation had a relative volume of 1.55% (0.512 L) with respect to the 33-L saline cuboid.

The total signal (2.5 V peak-to-peak; Figure 4) of the voluminous object is depicted by black dots. The green dotted curve shows the simulated total signal. The difference in amplitude between the measured and simulated total signals was similar in magnitude to the differential signals shown (red and blue) at a value of about 120mV. This residual deviation probably occurred due to geometric mismatches between real techniques and the idealized forward model and was partly influenced by additional coupling paths, such as capacitive effects. 

Even small deviations in the physical construction of the setup, such as slight shifts of one of the exciter strips, can lead to distortion of the total signal (Appendix B). However, these deviations had much less impact on the differential signals. Therefore, only differential MIT is presented here at this point. Both the red and the blue signal curves resulted from the difference between a single measurement with and without perturbation. The red curve (about 190 mV peak-to-peak) showed the signal behavior of the near-surface position, face-centered at the side wall, and the blue curve (about 130 mV peak-to-peak) showed that at the center of the volume (cf. Figure 2a). It is noteworthy that the relative signal of the central perturbation (5.2% of the total signal) was distinctly higher than the RV of the perturbation (1.55%). In previous MIT systems, the relative signal was usually much smaller than the RV, making small central perturbations virtually undetectable.

Environmental influences (e.g., motion artifacts, mechanical noise, EMI, etc.) led to a total noise signal of up to 2 mV peak-to-peak in differential signals. The signal-to-noise ratio (SNR) between the total signal (2.5V peak-to-peak) and the overall noise was thus close to 62 dB. The SNR given here, therefore, does not refer to the much stronger primary field, which is actually canceled out. Instead, it refers to the much smaller secondary field, equivalent to the imprint of the test body itself. To determine the electrical noise of the circuitry alone, a measurement was made without the test object and the carrier box. The electrical noise signal was 0.5 mV peak-to-peak. This results in a SNR of 74 dB between the total signal of the test object and the electrical noise.

**Figure 5:**
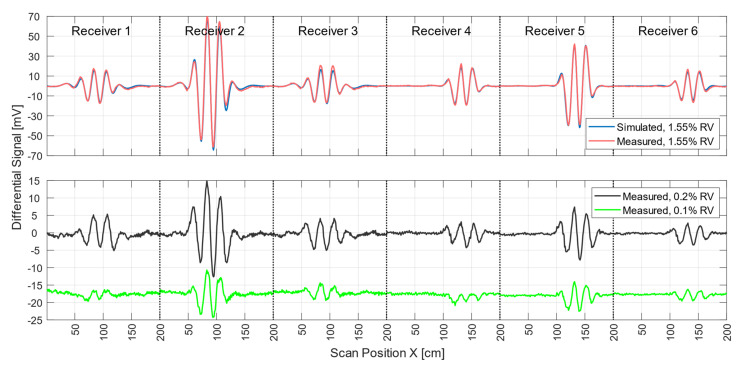
Differential scan signals from all six receivers from central perturbations with different RVs when comparing the incorporated perturbation and the whole test body. In the upper graph, the measured differential signal (red) of a perturbation with 1.55% RV overlapped with the simulation (blue) of the same arrangement. The lower graph shows the scan signal of a small non-conducting sphere with 0.2% RV (black) and a smaller sphere with 0.1% RV (green).

During each MIT measurement procedure (scan), the measurement signals of all six receivers were acquired. To obtain successful pattern reconstruction in the computer, these experimental signals should properly match the signals calculated by the forward computation of the scenario. The red and blue curves in Figure 5 show differential signals from all six receivers for real and simulated measurements of a non-conducting perturbation (1.55% RV) at the center of the volume, respectively. Other perturbation positions (calculated and measured) are shown in Appendix A. The blue signal curve was computed, and the red signal curve was the differential measurement obtained from two single measurement signals taken within 10 s of each other. The maximum deviation between the simulated and the measured curve was 6.2 mV and was recorded by receiver 2. The average deviation was 0.86 mV. Deviations between the practical measurements and the forward calculation thus have less effect on the differential signals than on the total signals. The smallest perturbation in the center of the body with a differential signal clearly above the noise floor (Figure 5, black line) was a sphere 5 cm in diameter at 0 S/m (65 mL, 0.2% RV; cf., Figure 2b). Smaller spheres at the center of the volume (33 mL, 0.1% RV; Figure 5, green line) resulted in critically weak signatures at a few mV (approaching −60 dB with respect to the total signal) that were more affected by motion artifacts and other noise, which ultimately prevented adequate evaluation.

### 3.2. Reconstruction in 3D

Experimental arrangements were tested in which the weakly conductive perturbations were located at positions that were previously difficult to detect with MIT setups and have, therefore, not yet been successfully reconstructed with practical measurements. Relevant examples are a perturbation in the central area of a voluminous body or two inner perturbations with complementary contrasts (i.e., higher and lower conductivity than the background) that were close together in the central area.

**Figure 6:**
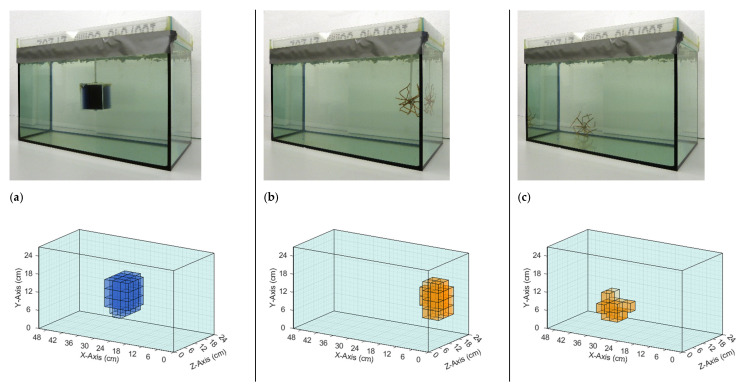
Comparison of real test body setups with the resulting reconstructions based on measured data. The background has a conductivity of 0.5 S/m in all three setups. (**a**) The perturbation with 0 S/m is located in the center; (**b**) The perturbation with 1 S/m is face-centered at the right side; and (**c**) The perturbation with 1 S/m is located at the bottom of the test body in the direction of the receiver. Note that the finer wire star shown in Figure 3c was used in the measurements and reconstructions. The coarser wire star in the photographs is used here only for better visibility.

**Figure 7:**
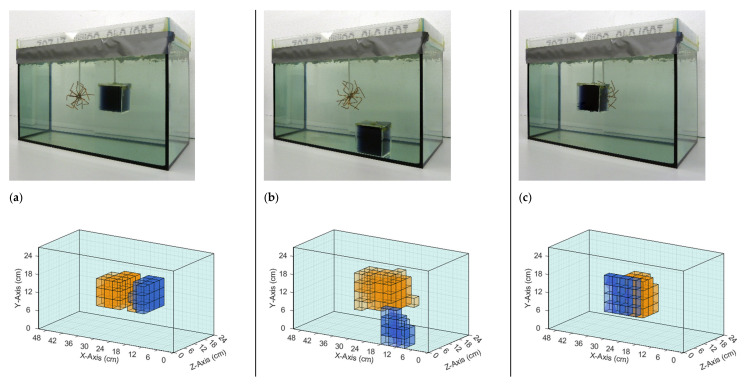
Comparison of real setups with the resulting reconstructions based on measured data. The background has a conductivity of 0.5 S/m in all three setups. (**a**) The perturbations with 0 and 1 S/m are centered in the body and displaced only in the x-direction; (**b**) The perturbations with 0 S/m are located at the bottom and in the direction of the exciter, and the perturbation with 1 S/m is in the center; (**c**) The perturbation with 1 S/m is in the center, and the perturbation with 0 S/m has same x- and y-position and is displaced in the z-direction towards the transmitter. Note that the finer wire star shown in Figure 3c was used in the measurements and reconstructions. The coarser wire star in the photographs is used here only for better visibility.

The iterative reconstruction algorithm (Section 2.4) recalculated and visualized the perturbations in the given homogeneous saline bath. Figure 6 and Figure 7 illustrate the reconstructions of different conductivity setups based on real measurement data. The top row in each figure shows the real setup, that is, the saline bath (33 L, 0.5 S/m) with the immersed perturbations. The bottom row shows computer reconstructions without prior knowledge regarding the probable locations or the character of the perturbations. The results are based on the numerical difference of two single measurements, with and without perturbations (Figure 4). The coloration mirrors the conductivity: the light blue, transparent background has a conductivity of 0.5 S/m; orange voxels have a larger conductivity (maximum 1 S/m) than the background; blue voxels have a smaller conductivity (minimum 0 S/m); and the lower the transparency, the greater the deviation from the background. The non-conductive perturbations shown as blue objects in Figure 6 and Figure 7 could represent over-inflated lungs. Perturbations with slightly increased conductivity (1 S/m) connected to the surrounding environment on all sides, shown as orange objects, could represent a bleeding mass or free fluid in the body. Note that the wire star shown in Figure 3c was used in measurements with a perturbation of 1 S/m. The wire star used in Figure 6 and Figure 7 with much thicker, colored wires was only used in the photos for better visualization.

The test body was discretized into 1224 voxels, each measuring 3 cm × 3 cm × 3 cm. A smaller voxel would have increased the computational effort disproportionately. Moreover, it is not yet possible to resolve very small conductivity modifications (RV < 0.2%), because they are affected too much by environmental influences. Due to the simplified forward problem (cf., Section 2.4) and the voxel size adjustment, the reconstruction in MATLAB using a standard office laptop without accelerating hardware took about three minutes. Therefore, it is reasonable to state that with more powerful yet affordable hardware and more effective coding, reconstruction could be achieved in a few seconds or even in real time instead of a few minutes.

## 4. Discussion

All of the MIT hardware presented here was designed to achieve the highest available sensitivity for the central area, ultimately resulting in more homogeneous sensitivity throughout the test body. Thus, signal amplitudes from perturbations near the surface or in the central areas were almost similar (cf. Figure 4). In the setup presented here, the relative amplitude of the central perturbation (130 mV peak-to-peak) corresponded to about 5.2% of the total signal (2.5 V peak-to-peak) and was thereby even higher than the RV of the perturbation (1.55%). An object near the surface (190 mV peak-to-peak) resulted in a signal at about 7.6% of the total signal. In addition, smaller voids (0.2% RV; Figure 5) in the central area were also clearly distinguishable from the noise floor (62 dB SNR). For comparison, previous practical studies usually used perturbations with an RV of 3 to 8 % of the total volume. These are thus usually more than twice as large as the conductivity perturbations (1.55% RV) reconstructed here in 3D and 15 times larger than the smallest clearly measurable perturbations (0.2% RV). Moreover, even such a small perturbation delivered a relative signal (1.1%) that was proportionally larger than the RV (0.2%). Overall, a specific gain of about 4 was achieved with the undulator MIT setup used here with its lateral scan procedure. Due to the topology of the eddy current distribution and receiver design, the overall signal of the whole object (Figure 4, black curve) was intentionally reduced with respect to the signal of a central perturbation (Figure 4, blue curve), which proportionally reduced the dominant dislocation noise [8]. These effects could be exploited in conductive volumes extending in all directions and not necessarily just in regular cuboids, making the technique promising for, for example, the human torso.

Another fundamental requirement for accurate reconstruction is good agreement between calculated and measured signal characteristics (Figure 5). The differential signals obtained here matched almost perfectly (with an average deviation of 0.86 mV) and thereby confirmed the applied forward theories and the chosen discretization. Combined with the higher sensitivity for the central areas, it was possible for the first time to reconstruct a weakly conductive volume with low-contrast inhomogeneities throughout its depth (Figure 6 and Figure 7) based on measurement data quickly acquired using experimental MIT. The absolute signal, however, still showed a significant mismatch (maximum 120 mV) between forward theory and measurement, presumably due to geometric mismatches of the undulator (Appendix B). Such mismatches were in the order of an included perturbation and currently prevent MIT using absolute measurements, that is, not based on differential measurements. There are several reasons why there might be a geometric mismatch between theoretical design and practical system. Since this is only an experimental setup to prove the basic methodology, it is implemented with a wooden frame. The wood can deform, for example, due to changes in temperature or humidity. Furthermore, the two narrow outer conductor strips (6 mm) of the undulator (Figure 1d) were handmade, which can lead to minimal differences in width, resulting in a small but noticeable current difference and imbalance throughout the undulator. In the future, the structure should be supported by a rigid fiber composite material and the elaborated dimensions of the undulator setup should be manufactured by machine.

The reconstructions shown in Figure 6 demonstrated that individual conductivity deviations (± 0.5 S/m) were detected at different positions in the voluminous background. Both the surface positions (Figure 6b,c) and the central position (Figure 6a) were resolved, the former often being shown in typical MIT setups, while the latter, having no proximity to any outer surface, was typically omitted in previous publications. Due to the more homogeneous sensitivity resulting here, inhomogeneities at the center and at the edges were detected simultaneously (Figure 7b,c), which was previously not possible because of the much stronger signals near the surfaces, compared with the weak imprints from central areas. Furthermore, clear differentiation was demonstrated in the x-direction (Figure 7a), y-direction (Figure 7b), and z-direction (Figure 7c). In Figure 7b, the reconstructed conductivity perturbation in the center (1 S/m) seems to occupy more volume than in Figure 7a,c. This could result from the basic reconstruction-algorithms but is probably also due to the fact that in Figure 7a,c the complementary perturbation (0 S/m) is positioned closer to the center perturbation, thus forming a clearer edge.

## 5. Conclusions

This study has demonstrated the practical implementation of a novel 3D MIT sensor system for the biomedical regime. For this implementation, many conditions had to be considered (Section 2) so that sufficient agreement between forward theory and technical realization could be achieved. The agreement of the simulated and measured differential signals proved to be satisfactory for differential 3D MIT and sufficient for imaging with experimental measurements. However, the deviation of the total signals, although improved, still prevents 3D MIT from absolute measurements. Geometric mismatches between the theoretical design and the practical system could be the cause of the remaining deviations. 

The undulator MIT setup using a lateral scanning method, which has been presented here, improved the sensitivity in the central areas, resulting in a similar response to objects near the surfaces compared to those at the center of the volume. As a result, and for the first time, experimental 3D MIT has been presented here that is sufficient to detect and localize weakly conductive, small perturbations (>0.2% RV) throughout the depth of a voluminous body. The perturbations had conductivities in the same range as organic tissue (0–1 S/m) and were connected to the environment, which had a conductivity of 0.5 S/m. The test body dimensions and general conductivity were similar to those of a human torso. The results presented here move MIT a step closer to harmless, convenient, and quick human tomography. In addition, the measurement and reconstruction results shown here confirm the simulations shown in [8]. The future task is clearly to develop an absolute tomography of an arbitrarily shaped body, that is, not based on differential measurements alone, where the outer contour of the body could be independently determined (e.g., by optical methods) and transferred to a computer. Further adjustment and refinement of the hardware and software would further increase and stabilize the resolution.

## Figures and Tables

**Figure A1:**
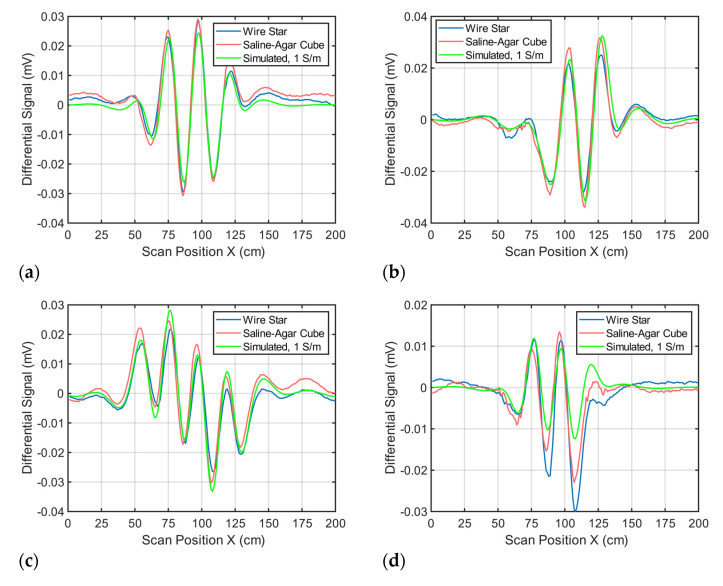
Comparison between saline-agar cube and wire star signal measurements. In addition, a simulated signal is added as a reference. (**a**) The perturbations are located in the center of the conductive background; (**b**) The perturbations are located face-centered at the side wall; (**c**) The perturbations are located close to the surface in the direction of the transmitter; and (**d**) The perturbations are located close to the surface in the direction of the receiver array.

**Figure A2:**
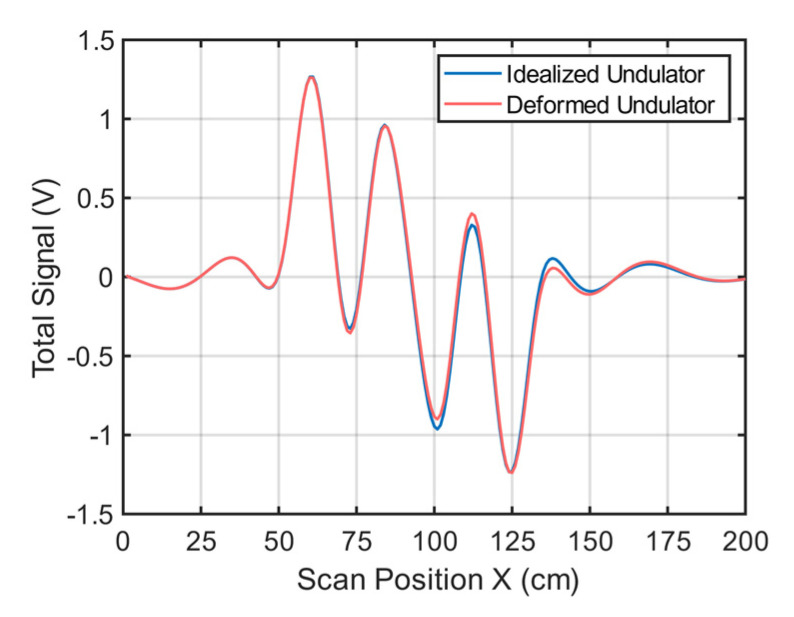
Simulated total signals. The blue line shows the simulated total signal of an undulator assumed to ideally match the intended geometries in Figure 1; The red line shows the simulated total signal with strip no. 8 of the undulator shifted by 1 cm in the z-direction.

## Data Availability

Not applicable.

## Appendix A

A saline-agar cube will dissolve and/or degrade over time, hindering experiments over longer periods of time. A much more durable wire star was used as an effective replacement. To verify equivalent signal behavior, the perturbations (saline-agar cube or wire star) were placed one after the other at different positions within the conductive background (body), and the differential signals of the perturbations were recorded (Figure A1). The signals show very similar behavior regarding amplitude and shape; therefore, a wire star can actually be used as a 0.512 L perturbation with 1 S/m.

## Appendix B

If the undulator in Figure 1 is only slightly deformed, this will influence the total signal. Figure A2 shows simulated signal changes (receiver 2) when one of the strips (here strip 8) is shifted by 1 cm in the z-direction. This slight change results in a maximum signal deviation of 74 mV. Similar changes occur for a slightly imbalanced current distribution in the undulator.
